# Differentially Expressed Genes of Virulent and Nonvirulent *Entamoeba histolytica* Strains Identified by Suppression Subtractive Hybridization

**DOI:** 10.1155/2014/285607

**Published:** 2014-09-17

**Authors:** Michelle A. R. Freitas, Ângela C. Alvarenga, Helen C. Fernandes, Frederico F. Gil, Maria N. Melo, Jorge L. Pesquero, Maria A. Gomes

**Affiliations:** ^1^Laboratório de Parasitologia, Instituto de Ciências Biomédicas, Universidade Federal de Uberlândia, Campus Umuarama, Uberlandia, MG, Brazil; ^2^Departamento de Parasitologia, Instituto de Ciências Biológicas, Universidade Federal de Minas Gerais, Avenida Antonio Carlos 6627, 31270-901 Belo Horizonte, MG, Brazil; ^3^Departamento de Fisiologia e Biofísica, Instituto de Ciências Biológicas, Universidade Federal de Minas Gerais, Belo Horizonte, MG, Brazil

## Abstract

*Entamoeba histolytica* is a parasite which presents capacity to degrade tissues and therefore has a pathogenic behavior. As this behavior is not shown by all strains, there have been several studies investigating molecular basis of the cytotoxicity process. Using the suppression subtractive hybridization (SSH) technique, differential gene expressions of two *E. histolytica* strains, one virulent (EGG) and one nonvirulent (452), have been analyzed with the purpose of isolating genes which may be involved with amoebic virulence. Nine cDNA fragments presenting high homology with *E. histolytica* previously sequenced genes were subtracted. Of these, four genes were confirmed by RT-PCR. Two coding for hypothetical proteins, one for a cysteine-rich protein, expressed only in the virulent strain, EGG and another one, coding for grainin 2 protein, exclusive from 452 strain. This study provided new insight into the proteins differences in the virulent and nonvirulent *E. histolytica* strains. We believe that further studies with these proteins may prove association of them with tissue damage, providing new perceptions to improve treatment or diagnosis of the invasive disease.

## 1. Introduction

Amoebiasis is a human infection caused by* Entamoeba histolytica, *a pathogenic and invasive parasite which kills about 100,000 individuals per year [1] in the world. Asymptomatic infections are produced by noninvasive amoebas. Symptomatic patients have invasive forms which are responsible for intestinal and extraintestinal changes [2] characterized by a large diversity of clinical situations. Intestinal amoebiasis may determine nondysenteric colitis, dysenteric colitis, amebomas, appendicitis, and extraintestinal amoebiasis, with the liver as the main affected organ.* E. histolytica *has a remarkable ability to destroy tissues, thus showing its pathogenic behavior. As this behavior is not common in all strains, molecular basis of the cytotoxicity process has been largely investigated [3–8]. Studies on the analysis of gene expression and differentiation in amoebas isolated from different clinical cases may contribute to a better understanding of the parasite's biology. There are many methodologies used with this purpose, and new techniques of differential gene expression have been described [6, 9–12]. Nevertheless, the suppression subtractive hybridization (SSH) technique, which is based on the identification of genes expressed only in cells and tissues of interest, still has not been used in order to identify genes which are possibly involved in the virulence of amoebas. SSH combines cDNA hybridizations with polymerase chain reaction (PCR) and enables high level-expressed genes not to be cloned and genes which are specific to certain structures, generally low expressed, to be selected [9, 13], contributing to characterize novel genes which may determine virulence, resistance to drugs, and infectivity. SSH has been used in order to evaluate differential gene expression between two* E. histolytica *strains, one virulent and another nonvirulent.

## 2. Material and Methods 

### 2.1. *E. histolytica* Strains

Two strains of* E. histolytica*, EGG, and 452 isolated in Brazil and maintained in axenic culture in TYI-S-33 medium [14] were selected. EGG was isolated from a patient who had dysenteric colitis and hepatic amoebiasis and strain 452 was isolated from an asymptomatic individual. Strains were characterized for virulence both* in vitro* and* in vivo*.

### 2.2. Inoculation in Hamster Liver

Eight male hamsters (*Mesocricetus auratus*), with one month old were used to each strain, EGG and 452. They were anesthetized with sodium pentobarbital (30 mg/kg) and the inoculation was carried out through a laparotomy procedure with an inoculum of 1 × 10^6^ trophozoites per animal. Six days after the inoculation, animals were sacrificed and opened for macroscopic examination of the liver. Animal injuries were classified from grade 0 to IV according to Diamond et al. [15]. This project was approved by the Animal Ethics committee of the Universidade Federal de Minas Gerais-CETEA (ETIC 158/06).

### 2.3. Cytopathic Effect

The interaction of trophozoites with Chinese hamster ovary cells (CHO) was as previously described with minor modifications [16]. For cell culture, 24-well plates were seeded with 1 × 10^5^ cells/well in Ham's F-12 medium with L-glutamine and supplemented with 5% fetal bovine serum. After cell growth for 48 hours at 37°C in an environment of 5% CO_2_, 1 × 10^5^ trophozoites were added to each well and incubated for 50 minutes under the same conditions as previously mentioned. After this time, cells that remained in the plate were fixed with 4% formaldehyde and stained with 0.1% methylene blue. Stain incorporated into cells was extracted with 0.01 M HCl. Aliquots of 100 *μ*L of each well were transferred to ELISA plates and absorbance determined at 660 nm in a microplate reader (Bio-Rad, Model 3550). The intensity of color extracted from monolayers of CHO cells which have not interacted with trophozoites served as control (0% of destruction). Experiments were carried out in duplicates and repeated five times. Statistical analysis was carried out with the nonparametric test ANOVA ONE-WAY, with a level of significance of 5% (*P* < 0.05).

### 2.4. Suppression Subtractive Hybridization

RNA was extracted with the use of the Trizol (Invitrogen) system following the manufacturer's instructions and RNA samples were submitted to Dnase to remove traces of DNA. The* PCR-select cDNA subtraction kit* (Clontech) was used in order to obtain differentially expressed transcripts [17] according to the manufacturer's instructions. Subtractions were carried out from EGG and 452 samples. Fragments of cDNA subtracted were extracted from gel, purified by Wizard SV gel kit and PCR clean-up system (Promega), and then cloned into pGEM-T easy vector (Promega) according to the manufacturer's instructions. Cloning was carried out using* Escherichia coli* bacteria DH5α (Life Technologies). Individual colonies were grown in 100 *μ*L LB-ampicillin at 37°C, plasmid was isolated, and the presence of the insert was confirmed by digestion reaction using* Eco*RI (5 U) followed by a PCR using nested primers (Clontech). For those clones presenting insert, plasmidial DNA was isolated from bacterial cultures using Wizard* plus* SV* minipreps* (Promega) kit. Positive clones were sequenced in accordance with a method previously described [18], making use of* DYEnamic ET dye terminator kit *(MegaBACE, GE Healthcare).

### 2.5. RT-PCR

Aliquots containing 3.0 *μ*g total RNA of EGG and 452 samples were used as a template for cDNA production using GE Healthcare system of reverse transcription (RT). Three microliters of RT product were used as a template in PCR containing specific primers (10 *μ*M) built from sequences subtracted from SSH (Table 1). Specific primers for a 300 bp fragment of *β*-actin gene were used as normalizer of the amount of RNA used in the reaction. Amplification products obtained by PCR were analyzed by electrophoresis in polyacrylamide at 5% silver stained.

### 2.6. Protein Polyacrylamide Gel Electrophoresis

Aliquots of supernatant homogenate containing 20 *μ*g protein were submitted to a 10% sodium dodecyl sulfate polyacrylamide gel electrophoresis (SDS-PAGE). Proteins migrated in the gel at room temperature and at a constant tension of 100 V. The electrophoresis running buffer was 20 mM tris-glycine (pH 8.3). Gels were stained by silver nitrate. Briefly, the proteins were fixed in the gel with a solution containing 40% methanol and 10% acetic acid followed by a 4% glutaraldehyde solution. After washing with water, the gel was emerged in a 0.4% silver nitrate solution. The proteins were then visualized by a developing solution composed by 5.7 × 10^−4^ M citric acid and 0.1% formaldehyde. The developing process was interrupted by a 1% acetic acid solution.

### 2.7. Analysis of Sequences

Partial sequences of cDNA were submitted to analysis using a BLAST 2.0 (basic local alignment search tool) server from the National Center for Biotechnology Information (NCBI) of National Library of Medicine of NIH (National Institute of Health), Maryland, USA, and from available database referring to* E. histolytica* Omniblast server (Sanger Institute). Translation of cDNAs and identification of possible domains were carried out using ScanProsite server (http://ca.expasy.org/).

## 3. Results

### 3.1. *In Vitro* and* In Vivo* Virulence

**Figure 1:**
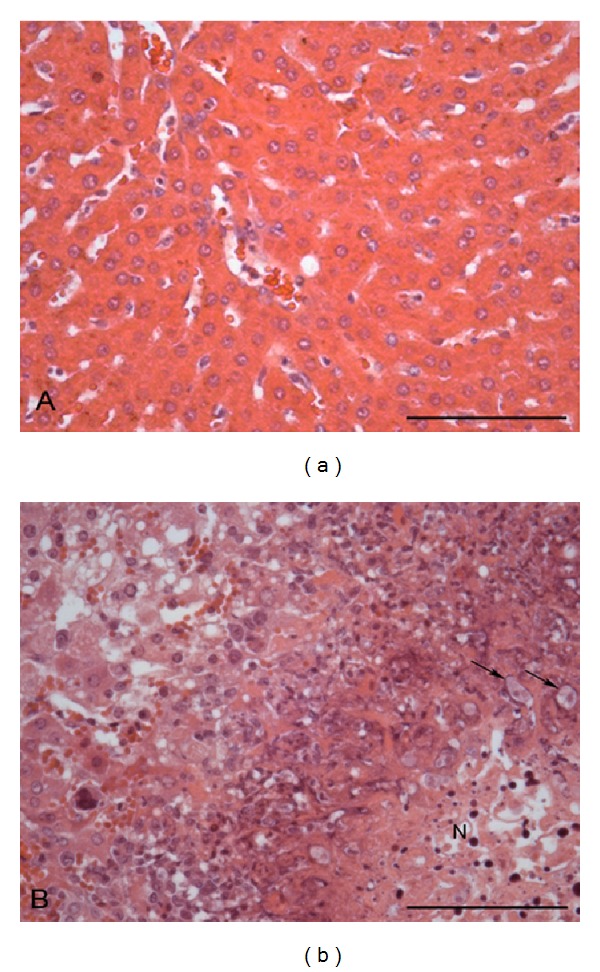
(a) Hamster liver inoculated with 452, a non-virulent strain of* E. histolytica* showing normal mucosa. HE 400X. (b) Hamster liver inoculated with EGG, a virulent strain of* E. histolytica* showing intense colliquative necrosis of hepatocytes (N). Trophozoites are shown (arrows). HE 400X.

*Inoculation in Hamster Liver*. The strain 452 did not cause lesions (grade 0) and the EGG strain presented virulence with grades varying from III to IV. Lesions presented a yellowish aspect and areas of parenchymal destruction substituted by necrosis (Figure 1).

**Figure 2:**
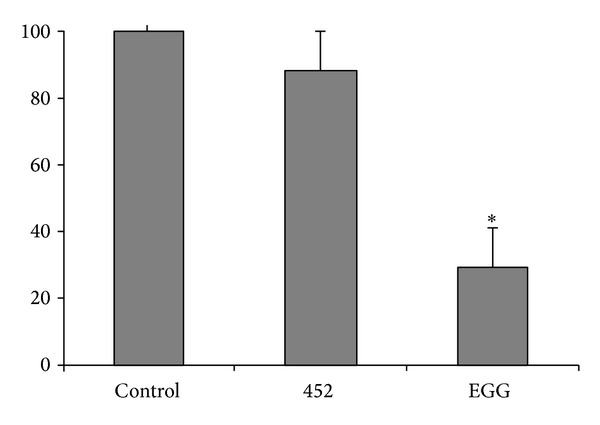
Percentage of CHO cell monolayers after incubation with* E. histolytica* trophozoites. Statistical analysis was carried out with the nonparametric test ANOVA ONE-WAY, with significance level of 5% (*P* < 0.05).

*Cytopathic Effect*. EGG strain destroyed 71% of cells in monolayers, being significantly more virulent (*P* < 0.05) than 452 strain which destroyed only 12% of cells (Figure 2) as compared to the control.

### 3.2. Suppression Subtractive Hybridization

**Figure 3:**
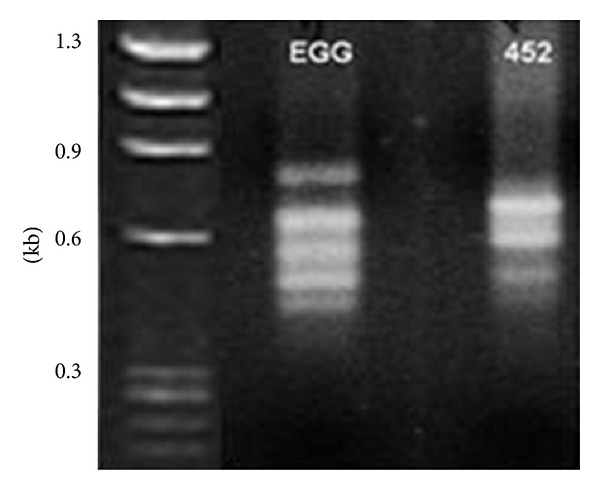
Agarose gel (0.8%), stained with ethidium bromide, showing products digested with Rsa-I after the subtraction process for EGG and 452 strains. M = molecular mass marker, φ-X174 DNA/HaeIII.

**Figure 4:**
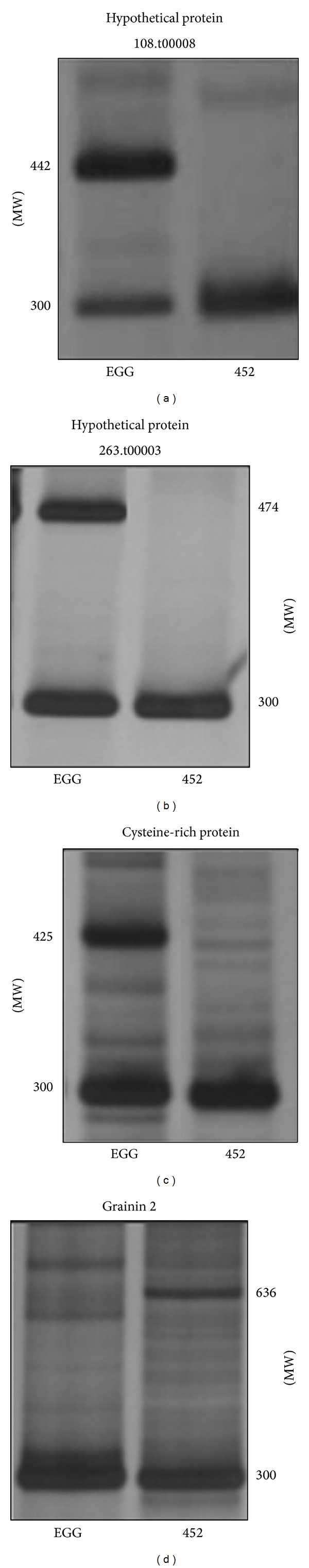
Polyacrylamide gel (5%) silver stained showing the expression of four differentially expressed cDNAs generated by the RT-PCR for each one of the proteins.

Two populations of cDNAs was obtained by the SSH technique and correspond to differentially expressed genes of EGG and 452 strains. The profile of bands obtained after subtraction reaction shows that five differentially expressed bands were observed for EGG and four bands for 452 strain (Figure 3). After sequencing, all the nine sequences showed homology to well-known genes of* Entamoeba histolytica*. From these, only four were confirmed by RT-PCR as differentially expressed, one from nonvirulent 452 strain and three from virulent EGG strain (Figure 4). The differentially expressed sequence obtained from the nonvirulent strain corresponds to the gene of protein grainin 2 (XP_650371.1) and the sequences obtained from the virulent strain correspond to two hypothetical proteins 108.t00008 (XP_652703.1) and 263.t00003 (XP_649888.1) and a cysteine-rich protein (XP_654688.1).

**Figure 5:**
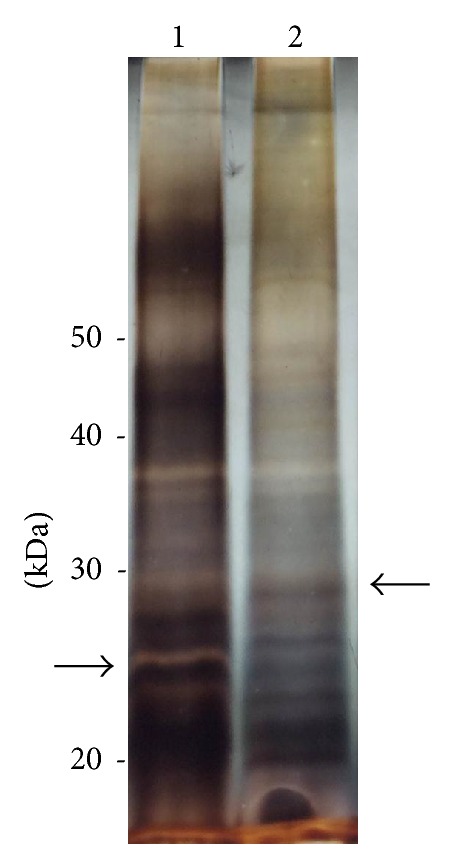
Electrophoretic profile obtained in a 10% polyacrylamide gel of 452 (lane 1) and EGG (lane 2) homogenate strains. Arrows shows differentially expressed bands of ~25 kDa (left) and 27 kDa (right).

The electrophoretic profile obtained for crude homogenate of the two strains is shown in the Figure 5. It can be observed, in the sample derived from strain 452 (lane 1), some differentially expressed bands presenting molecular mass between 20 and 30 kDa, especially one of ~25 kDa that might be related with grainin 2. On the other hand, the sample derived from strain EGG (lane 2) shows a ~27 kDa band, which could be related to the cysteine-rich protein.

## 4. Discussion

It is clearly shown in the literature that subtractive hybridization (SH) and particularly suppression subtractive hybridization (SSH) have been used as a suitable tool for experimental identification of novel genes in eukaryotes as well as prokaryotes, whose genomes have been sequenced, or the species whose genomes are yet to be sequenced. The validity of this technique in identifying genes related to virulence in microorganisms is supported by several studies [19–21], including Janke et al. [22] who found 22 genes specific for the 536 pathogenic strain of* Escherichia coli* when compared to the nonpathogenic strain K12-MG1655.

With the purpose of identify genes and putative related proteins involved in the amoebiasis physiopathology, the SSH technique was used in two* E. histolytica *strains, one isolated from a patient with hepatic amoebiasis and another from an asymptomatic carrier. At first, strains were characterized, as for* in vivo *and* in vitro* virulence, by inoculation into hamster liver and cytopathic activity. These methods are widely used for virulence characterization of* E. histolytica* strains [3, 23–27]. EGG strain produced important lesions in the animals inoculated while in contrast 452 strain did not infect animals, corroborating to the clinical form of patients whose isolated were obtained. Concordance was also observed in* in vitro* assays in which EGG strain showed to be able to destroy cells more significantly than 452.

By the SSH technique, it was identified, nine cDNA fragments presenting high homology with previously sequenced genes from* E. histolytica *genome [28]. Among the identified differentially expressed genes, some are involved with several physiological processes such as metabolism, endocytosis, protein biosynthesis, signal transduction, and others with an unknown function. Among these, four genes were confirmed by RT-PCR as differentially expressed. Among them are two hypothetical proteins and one cysteine-rich protein, expressed in the EGG strain, and the protein grainin 2 expressed in the 452 strain. Grainin 2, and grainin 1 are found in granules of* E. histolytica*, and has recently been described as calcium-binding proteins with unknown functions[29]. Although several functions are hypothetically attributed to these proteins, the most likely probably are controlling endocytosis pathways and granule discharge depending on calcium concentration. Corroborating our results, a recent study showed that these proteins are expressed in high levels in* E. histolytica* strains with reduced virulence [3]. In addition, it was found a low level of grainin 1 expression in HM1 strain recently isolated from hamster liver, when compared to that maintained for a long time in a culture medium [3]. Afterwards, reduced levels of grainin 1 and 2 were reported in HM1 trophozoites isolated from mice colon [30] and by comparing trophozoites of human colon to those maintained in culture medium [31]. In our studies, the protein grainin 2 seems to be expressed only in the non-virulent 452 strain. Therefore, considering our results and the others, obtained by different techniques, showing a relationship between grainin 2 levels and virulence, we may hypothesize that this protein may be associated with reduced virulence, thus serving as a marker for evaluating the pathogenic potential in* E. histolytica*.

From among differentially expressed genes in the EGG strain, we have found that one codes for a cysteine-rich protein. Currently, it is known that there are two groups of kinase receptors rich in cysteine with domains containing CXC and CXXC repeats [28]. Membrane receptors play an important role in parasite-host interactions and account for tissue adhesion and destruction [6, 8, 12, 32, 33]. This cysteine-rich protein is likely to be a kinase receptor which may contribute to the amoebic system of cell invasion and destruction [30].

Two other cDNA fragments, obtained from EGG strain presented homology with* E. histolytica* genes which code proteins with yet unknown functions. With the sequencing of* E. histolytica* genome, it was demonstrated that, among protein-coding genes of this parasite genome, 41% correspond to hypothetical proteins, with functions which have not been characterized yet [28]. The role of these proteins in the virulence of amoeba needs to be determined. However, as they have been found only in virulent strain, these proteins may contribute to a successful tissue invasion by the amoeba or for their survival in the injured tissue.

## Figures and Tables

**Table 1:** Primers used for RT-PCR.

	Protein	GenBank number access	Primer 1	Primer 2
1	Actin		GCTGCATCAAGCAGTGAA	GAATGATGGTTGGAAGAG
2	Grainin 2	XP_650371.1	GTCTTTGTTTGCTATCC	GTTCTTGCACTTTCAGG
3	Rab7-GTPase	XP_649196.1	TGCAGGGAAAAAGATTC	AACAAGGGCAATCAGAC
4	Rib. 60S	XP_655273.1	ACTTCACATTAGCCCAG	TAATAGCAACCATACCG
5	Hip. 108.t00008	XP_652703.1	TCAGCATGTGCTCAATC	TTCCATGTCCAATTCTC
6	Hip. 263.t00003	XP_649888.1	TGGGTCTCTTCAGACAG	ATAACTTTTCCACCTCC
7	Cysteine-rich protein	XP_654688.1	TGTCAGGAACACCAATC	CTACAGAACTTTCCTCC
8	Rib 40S-S17	XP_657103.1	GGAGTCAGAACTAAAAC	AGCATTTTGAGAGTAAC
9	Eif-5A	XP_657397.1	AATGCTGAACATTCTGG	GCTTCAATACCCATAGC
10	Gal/GalNac	XP_656181.1	GCAGGACAAGGACAAGTTG	GATCTGCTTCACAATTAGC
